# Effect of Acupuncture on Chronic Pain with Depression: A Systematic Review

**DOI:** 10.1155/2020/7479459

**Published:** 2020-06-25

**Authors:** Bin Yan, Shibai Zhu, Yu Wang, Gula Da, Guoqing Tian

**Affiliations:** ^1^Department of Traditional Chinese Medicine, Peking Union Medical College Hospital, Chinese Academy of Medical Sciences, Beijing, China; ^2^Department of Orthopedic, Peking Union Medical College Hospital, Chinese Academy of Medical Sciences, Beijing, China; ^3^Department of Nursing, Peking Union Medical College Hospital, Chinese Academy of Medical Sciences, Beijing, China; ^4^Department of Rheumatology and Immunology, Peking Union Medical College Hospital, Chinese Academy of Medical Sciences, Beijing, China

## Abstract

**Background:**

Numerous studies suggested that chronic pain and depression were closely related and widespread in the population. When patients have symptoms of chronic pain and depression, the corresponding treatment will become difficult. Acupuncture, a unique therapeutic method of traditional Chinese medicine, has been reported to potentially serve as an alternative treatment for patients with comorbid chronic pain and depression by many research studies.

**Methods:**

A comprehensive search was conducted through the online database, including the Cochrane Library, PubMed, EMBASE, SinoMed, CNKI, and Wanfang database. Trials were RCTs published in the English or Chinese language, recruiting participants with chronic pain and depression comorbidity. The primary outcomes were the Visual Analogue Scale (VAS) and Hamilton Depression Scale (HAMD). Statistical analyses were conducted using Review Manager 5.3. Each trail was quality appraised with the five-point Jadad Score.

**Results:**

7 eligible RCTs involving 535 patients were included. Better therapeutic effect and safety could be observed in the experimental group compared with the control group. There was a significant decrease in the VAS (mean difference (MD) = −0.68 (−1.24, −0.12), *P*=0.02) and HAMD (MD = −2.18 (−3.09, −1.26), *P* < 0.00001) scores and the incidence of adverse events between two groups.

**Conclusion:**

In the treatment of chronic pain with depression, acupuncture could not only get better clinical efficacy, but also have higher security compared with medicine therapy, which can be used in patients with poorer response to the conventional medication or suffering from serious side effects.

## 1. Introduction

According to the definition of chronic or persistent pain given by the International Association for the Study of Pain, if the pain lasts longer than 3 months or beyond the time period when an acute insult would have been expected to heal, it becomes a chronic condition [1]. Chronic pain is considered one of the most prevalent physical conditions in developed countries, affecting approximately 1 in 10 adults [2]. Since pain is “an unpleasant sensory and emotional experience associated with actual or potential tissue damage, or described in terms of such damage,” depression and pain always co-occur [3]. Additionally, the physiological and emotional burden of chronic pain and a lack of efficient treatments might act as barriers to recovery and contribute to the development of persistent pain and major depression [4].

Depression is prevalent around the world, affecting more than 350 million people worldwide [5]. A recent up-to-date article noted that depression is the most common psychiatric disorder in the general population and the most common mental health condition in patients seen in primary care [6]. It is also a leading cause of disability and can cause high levels of distress and increased risk of suicide [7]. The World Health Organization (WHO) projected that depression will rank the largest burden of disease worldwide by 2030 [8]. In practice, for the diagnosis of depression, the two main classificatory diagnostic systems, the Diagnostic and Statistical Manual of Mental Disorders and the International Classification of Diseases, rely on the identification of some key symptoms [9]. Therefore, depression is a disorder with symptoms forming a syndrome and causing functional impairment, which can lead to considerable loss of productivity and quality of life.

As two of the most widespread disorders, studies suggested that pain and depression were closely related [10]. The combination of these two disorders can exacerbate the experience of one's health state, interfere with people normal functioning, and decrease the quality of life seriously [11]. A substantial proportion (30% to 45%) of patients with chronic pain present with frank symptoms of depression [12], and also 52% to 65% of patients with depression suffer from chronic pain [13]. Mental distress contributes to more serious pain, at the same time to greater pain-related disability and poorer response to the pharmacological treatment [5, 14]. In other words, chronic pain and depression create a vicious cycle in which pain worsens symptoms of depression, and then the resulting depression worsens feelings of pain. Studies have shown that depression and chronic pain share some of the same neurotransmitters and nerve pathways [15]. However, the causal association between depression and chronic pain is yet unclear.

In view of the biopsychosocial aspects involved in chronic pain, a multimodal approach to management is essential [16]. This usually involves pharmacological or nonpharmacological therapy or both. Many guidelines [17, 18] recommended acetaminophen as the first-line agent for chronic pain due to its safety and tolerance. However, it is not an ideal choice for chronic inflammatory pain (such as rheumatoid arthritis and osteoarthritis) compared with nonsteroidal anti-inflammatory drugs (NSAIDs) due to the lack of anti-inflammatory activity [19]. COX2-selective and nonselective NSAIDs are particularly helpful in treating an inflammatory type of pain, which display both analgesic and anti-inflammatory properties [20]. However, the side effects also need to be vigilant, especially the gastrointestinal and cardiovascular events [21, 22]. Generally, from a pharmacological perspective, opioids are considered as the most powerful painkillers [23]. In addition, these medications have been increasingly used for the treatment of chronic nonmalignant pain which failed response to other medications [24]. However, previous reports also have indicated the prescription opioid misuse [2, 25] and death [26] in the comorbidities of the joint drug problem. Other medications, including anticonvulsants, serotonin-norepinephrine reuptake inhibitors (SNRIs), antidepressants, benzodiazepines, muscle relaxants, and topical lidocaine, all have their own indications and adverse reactions [27]. Thus, the points on the management of chronic pain have shifted to a multimodal and multidisciplinary therapy, and it also includes psychological (mindfulness meditation and cognitive behavioral therapy), rehabilitative, interventional, and complementary/alternative therapies [27–30].

Conventional treatment of depression mainly includes medication, psychotherapy, and physiotherapy. Taking antidepressants as the most preferred treatment of this disease, only a third of patients with depression respond fully to antidepressant medication [31]. Long-term side effects and drug dependence make patients less compliant with them. Although evidence-based studies confirmed that cognitive behavioral therapy (CBT) was effective [32], its effect was a gradual and cumulative process, which was slower than that of drugs. Therefore, patients are often more willing to accept drug treatment than to tolerate the gradual process in the early stage. Physical therapy includes electroconvulsive therapy (ECT), vagal nerve stimulation (VNS), and transcranial magnetic stimulation (TMS). Although the safety and efficacy of physical therapy have been verified by some studies [33–35], the effectiveness varies from person to person [36]. Therefore, for some special types of depression, such as chronic, refractory, severe, and adolescent, the combined treatment model has become a new trend of depression treatment [31, 34]. In brief, medication, psychotherapy, and physiotherapy have been shown to be effective, but the actual clinical effect is unsatisfactory.

In terms of management for chronic pain and depression comorbidity, there was a significant overlap in the pharmacological treatment [28, 37]. However, these medicine cotreatments may induce new clinical issues due to drug-to-drug interactions or drug-related adverse events. For instance, application of opioids can relieve chronic pain effectively, but it was agreed that it can also cause severe dependence and addiction in patients [38], and long-term use of opioids has been confirmed to increase the risk of depression [39]. Benzodiazepines, a kind of antidepressants, do not have analgesic effect; however, up to one-third of patients taking opioids for chronic pain have reported taking benzodiazepines simultaneously, which may increase the risk of sedation and respiratory depression [40]. Therefore, it is necessary to find another treatment method with good therapeutic effect and little side effects.

Acupuncture, a unique therapeutic method of traditional Chinese medicine with a history of thousands of years, has become a widely recognized alternative and complementary therapy in clinical practice [41]. Acupuncture uses needles to stimulate specified acupuncture points, which has the advantages of simple operation, economical cost and few side effects, and it has obvious curative effect on pain and depression separately. Many clinical research studies have verified that acupuncture is an effective treatment for patients with cancer pain, migraine, and low back pain [42–44]. There are also many randomized controlled trials having confirmed that acupuncture can alleviate depression and improve patients' quality of life [45, 46]. As the optimized comorbid chronic pain and depression management is to allow progress in restoring function while reducing long-term reliance on medical therapy [47], acupuncture may potentially serve as an alternative treatment for patients with comorbid chronic pain and depression.

To date, there have been several systematic reviews of acupuncture in the treatment for chronic pain or for depression [48, 49]. However, the effect of acupuncture as a treatment for chronic pain and depression comorbidity is questionable, and there is no systematic review of the use of acupuncture for comorbid chronic pain and depression, so the experts may confront problems to conduct further research in the related field. Therefore, a comprehensive systematic review of acupuncture in the treatment for chronic pain with depression is needed.

## 2. Methods

### 2.1. Search Strategy

A comprehensive search for studies about the effectiveness of acupuncture for chronic pain with depression was conducted through the online database. The following electronic databases were searched from inception to March 17, 2020: the Cochrane Central Register of Controlled Trials, PubMed, EMBASE, Chinese Biomedical Database (SinoMed), China National Knowledge Infrastructure (CNKI), and Wanfang database. The following terms were used: “chronic pain” OR “pain, chronic” AND “depression” OR “depressive disease” OR “depressive disorder” AND “acupuncture therapy” AND “random.” During searching Chinese databases, the similar search strategy with Chinese terms was adopted. The initial database search was done by 3 authors (Yan, Zhu, and Wang) independently to ensure reproducibility.

### 2.2. Inclusion and Exclusion Criteria

#### 2.2.1. Type of Study

Trials were eligible if they were randomized controlled trials (RCTs) recruiting participants with chronic pain and depression comorbidity, regardless of whether there was single blind, double blind, or nonblind.

#### 2.2.2. Type of Participant

Patients diagnosed with chronic pain combined with depression or depression combined with chronic pain will be included. The main concern of studies cited must be chronic pain and depression, that is to say, the author must explain the definition or diagnostic criteria for chronic pain and depression. There will be no limits on the age, sex, and source of cases.

#### 2.2.3. Intervention

Patients in the experimental group were treated with acupuncture alone or in combination with other therapy, while those in the control group were subjected to other therapy without acupuncture for chronic pain with depression.

#### 2.2.4. Outcome Measures

The primary outcomes of interest were the pain scores and depression severity, i.e., Visual Analog Scale (VAS): higher scores indicate more severe pain, and Hamilton Depression Scale (HAMD): higher scores indicate a greater degree of depression, and to observe the changes of indicators before and after intervention. The secondary outcomes included any adverse events, i.e., Treatment Emergent Symptom Scale (TESS) or Rating Scale for Side Effects (SERS).

#### 2.2.5. Exclusion Criteria

In order to evaluate the independent effects of acupuncture, the following trials were excluded: (1) conference abstracts, review articles, animal studies, cadaveric studies, in vitro studies, or articles published in a form other than clinical trials; (2) any control group that included acupuncture therapies; (3) literatures without relevant indicators or quantitative data; (4) evaluation indicators include only chronic pain or only depression; (5) repeated published literature.

### 2.3. Selection of Studies

4 authors (Yan, Zhu, Wang, and Da) independently screened all potential eligible studies. Titles and abstracts were first screened to exclude irrelevant papers. Full text of all articles of potentially relevant abstracts were retrieved and screened according to the study inclusion and exclusion criteria. Final article selection was done independently by all four reviewers, and disagreements were resolved by consensus.

### 2.4. Quality Assessment

4 authors (Yan, Zhu, Wang, and Da) independently conducted the methodological quality of all included studies. Each article was quality appraised with the five-point Jadad Score [50]. Three factors associated with risk of bias were evaluated: randomization, blinding, and follow-up. The specific scoring criteria are as follows: when the study provides a detailed description of randomization, such as using a random number table, 2 points are obtained; if only a random method is used and there is no exact description, 1 point is obtained; if there is no random allocation, no score. 2 points were scored when the study used the appropriate placebo or a similar method; 1 point was scored when the trial involved blinding and no description; no blinding means no score. When describing the follow-up and the reasons for loss of follow-up, score 1 point, otherwise no score. If the total score is greater than or equal to three, the paper is considered to be of high quality.

### 2.5. Data Extraction

Data were extracted into a prespecified data extraction table, with items including the authors' names, the year of publication, total sample size, age, gender, detailed intervention information of two groups, outcome measures, and adverse reactions.

### 2.6. Statistical Methods

Review Manager 5.3, provided freely by the Cochrane cooperation net, was applied for statistical analysis. The primary outcomes, VAS and HAMD, were both continuous variables, and mean difference (MD) was used as effect values. The confidence internal was set as *α* = 0.05.

## 3. Results

### 3.1. Results of the Search

**Figure 1:**
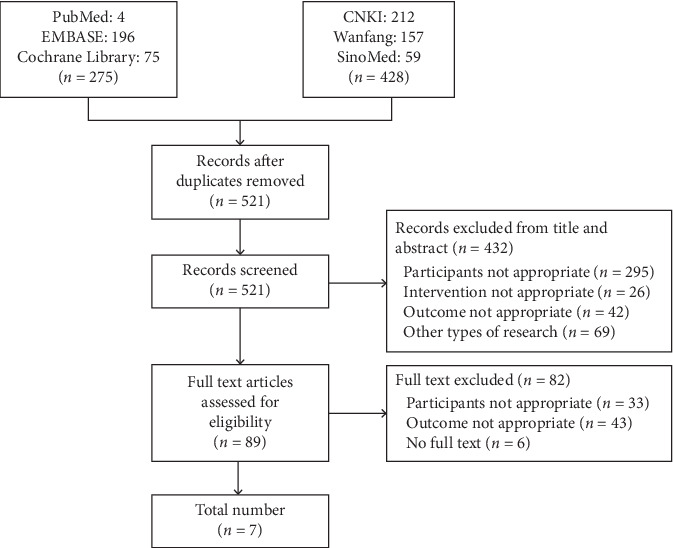
Study flow diagram.

A total of 521 references were retrieved after removing duplicates (Figure 1). 3 authors (Yan, Zhu, and Wang) independently screened these references. Based on the review of the title and abstract, 89 full-text papers were reviewed and 7 eligible RCTs [51–57] involving 535 patients were included. All 7 RCTs were conducted in China and were published between 2000 and 2018. There were a total of 265 participants receiving acupuncture alone or in combination with other therapy (experimental group) and 270 receiving other therapy without acupuncture (control group).  Table 1 shows the distributions of sex, age, and time since diagnosis between the experimental and control groups, and the detailed intervention information is shown in Tables 2 and 3.

### 3.2. Comparison of the Pain-Related Score

**Figure 2:**
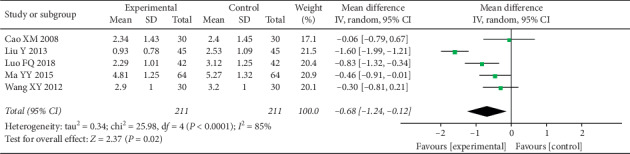
Forest plot depicting the VAS.

**Figure 3:**
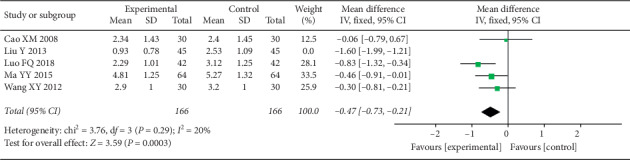
Forest plot depicting the VAS after sensitivity analysis.

A total of 6 studies assessed pain [51–56], and 5 of them used the VAS score [51, 53–56], while the other one used the comprehensive headache score [52]. The evaluation time point of each study was different, and most studies chose pretreatment and 4 weeks after treatment as time points for pain assessment (Table 4). There was no significant difference in VAS between the two groups before therapy. After 4 weeks of treatment, VAS decreased significantly in both groups, and the experimental group was more significant than the control group (MD = −0.68 (−1.24, −0.12), *P*=0.02, *I*^2^ = 85%) (Figure 2). However, there was a high degree of heterogeneity in the study (*I*^2^ = 85%). Sensitivity analysis reduced heterogeneity (*I*^2^ = 20%) (Figure 3) after deleting a study whose data were apparently different from the others.

### 3.3. Comparison of the Depression-Related Score

**Figure 4:**
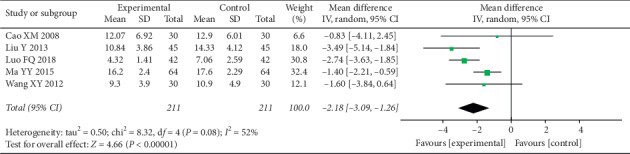
Forest plot depicting the HAMD.

**Figure 5:**
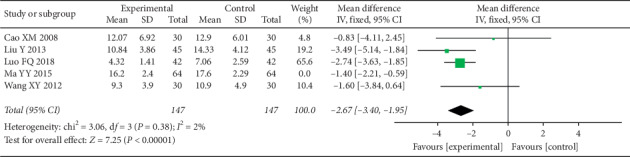
Forest plot depicting the HAMD after sensitivity analysis.

In the eligible RCTs, all trials conducted the depression-related assessments, and HAMD was used in 6 of them [51, 53–57]. Similar to the VAS, most studies included the time point of pretreatment and 4 weeks after treatment (Table 5). There was no significant difference in HAMD between the two groups before therapy. After 4 weeks of treatment, HAMD decreased significantly in both groups, especially in the experimental group, which indicates that there was a significant difference between the two groups (MD = −2.18 (−3.09, −1.26), *P* < 0.00001, *I*^2^ = 52%) (Figure 4). Similarly, because of the high heterogeneity, after removing a research, the heterogeneity was significantly declined (Figure 5).

### 3.4. Security Assessment

All studies recorded adverse events during treatment in both groups. Incidence of adverse events was recorded in 5 studies [51–54, 56], and other security assessments included TESS [53, 57] and SERS [55]. 2 studies reported no difference in the incidence of adverse events between the two groups [53, 54], and other studies have shown that acupuncture therapy could significantly reduce the incidence of adverse events and the adverse event-related score (Table 6).

### 3.5. Quality Assessment

The quality assessment of the trials was performed using the five-point Jadad Score (Table 7). Randomization was mentioned in all included studies, but only one had a detailed description of it [51]. Blind method cannot be used in research due to the characteristic of acupuncture. Besides, only two trails describe the details of follow-up [51, 52].

## 4. Discussion

This study is a comprehensive systematic review of acupuncture in the treatment for chronic pain with depression. There were significant differences in the VAS score and HAMD, decreasing between the two groups after treatment, indicating that acupuncture used alone or in combination with medication therapy can relieve pain and depression better than medication. Patients in the experimental group had lower incidence of adverse events and side effect scores, which proved the safety of acupuncture. The above results suggested that acupuncture has not only a better clinical result on the treatment of chronic pain with depression but also has a higher security compared with medication therapy.

When it comes to acupuncture therapy, we found that different individual studies might utilize different acupoints, but from these prescriptions we could also find something in common. In practice, there are many ways to select acupoints during acupuncture treatment. Selection of local acupoints around the pain location was the main method to treat pain. In the treatment of depression, acupoints on the head were selected more frequently, such as Governor Vessel (GV) 20, GV 24, Extra-points of Head and Neck (EX-HN) 1, and EX-HN 3. In addition, some distal acupoints were also be selected, including Pericardium meridian of hand-jueyin (PC) 6, Heart meridian of hand-shaoyin (HT) 7, Spleen meridian of foot-taiyin (SP) 6, and Liver meridian of foot-queyin (LR) 3. According to traditional Chinese medicine theory, human emotional activities are closely related to the five internal organs, so this explains why acupoints on multiple meridians can be selected for the treatment of depression.

The treatment of chronic pain and depression comorbidity has always been a tough problem because of poorer response to the pharmacological treatment and long-term side effects of drugs. Acupuncture, as an effective, simple, and economical treatment, has been used in the treatment of many diseases in China. The results of the systematic review suggested that acupuncture has a promising application prospect due to its unique advantages for the treatment of chronic pain with depression, and it can be used in patients with poorer response to the medication or suffering from serious side effects.

Previous systematic reviews of acupuncture for chronic pain and depression comorbidity are not available. However, findings from other chronic pain reviews and depressive disorder reviews are consistent with this review and indicate that acupuncture used alone or in combination with other treatment measures has a better therapeutic effect to the control group [49, 58–61].

There are many patients suffering from chronic pain with depression at present, and the findings of this systematic review provided a valuable alternative to these patients. However, most of the included articles had a quality score of no more than 2 points [52–57], and only 1 article got 3 points [51], which meant that the quality of the majorities was low. Considering the characteristics of acupuncture therapy itself, it is difficult to implement the blind method. The acupuncture points, frequency, and course of treatment selected in different studies were different, so there were difficulties in formulating a standardized treatment. Thus, more high-quality, rigorously designed, and well-controlled RCTs are still needed to support the clinical application of acupuncture for the treatment of chronic pain with depression.

The design of future studies in this area can play an important role in improving the quality of evidence and address the lack of evidence to support acupuncture for the management of chronic pain and depression comorbidity. Hence, there are some recommendations to guide future research: (i) improving methodological quality; (ii) extending follow-up periods to include intermediate- and long-term follow-up; (iii) increasing sample sizes. High-quality studies should also include costs, risks, and synergistic values of combining acupuncture with drugs compared with monotherapy otherwise.

## 5. Conclusion

Acupuncture has a promising application prospect due to its unique advantages for the treatment of chronic pain with depression comorbidity, which can be used in patients suffering from some certain chronic pain with depression comorbidity with poorer response to the conventional medication or suffering from serious side effects. High-quality RCTs are needed to support the current clinical application of acupuncture for the treatment of chronic pain with depression comorbidity and to broaden the clinical application.

## Figures and Tables

**Table 1:** Basic information of 7 RCT studies.

No.	Author	Year	Sample (EG/CG)	Age (SD)		Gender (EG/CG)	Time since diagnosis (SD) (EG/CG)
				EG	CG		
1. [51]	Wang	2012	60 (30/30)	51.5 (4.1)	50.3 (4.7)	—	—
2. [52]	Xiao	2015	48 (24/24)	53.17 (9.89)	50.58 (8.80)	8/16 (M/F)/10/14 (M/F)	—
3. [53]	Ma	2015	12 8 (64/64)	39.93 (12.93)	38.69 (14.19)	27/37 (M/F)/29/35 (M/F)	20.33 (12.76) m/20.00 (12.12) m
4. [54]	Luo	2018	84 (42/42)	57.15 (11.26)	57.39 (11.58)	22/20 (M/F)/23/19 (M/F)	42.31 (8.75) m/41.92 (8.32) m
5. [55]	Liu	2013	90 (45/45)	47 (8)	48 (8)	15/30 (M/F)/16/29 (M/F)	3.5 (1.8) m/3.2 (1.7) m
6. [56]	Cao	2008	60 (30/30)	20–70		23/37 (M/F)	0.5–30 y
7. [57]	Huang	2000	65 (30/35)	30.39 (7.01)		25/40 (M/F)	—

EG, experimental group; CG, control group; —, not available; m, month; y, year.

**Table 2:** Detailed intervention information 1.

No.	Study type	Diagnosis	EG	CG	Duration (wks)
1	RCT	Depression with chronic pain	Abdominal acupuncture	Deanxit	4
2	RCT	Migraine with depression	Acupuncture	Deanxit combined rizatriptan benzoate tablets	4
3	RCT	Depression with chronic pain	Acupuncture combined duloxetine	Duloxetine	8
4	RCT	Recurrent chronic trigeminal neuralgia accompanied by depression	Acupuncture combined traditional Chinese medicine	Traditional Chinese medicine	4
5	RCT	Depression with chronic pain	Acupuncture combined SSRI antidepressants	SSRI antidepressants	4
6	RCT	Chronic pain with depression	Acupuncture	Deanxit	4
7	RCT	Depression with chronic pain	Acupuncture	Amitriptyline	6

EG, experimental group; CG, control group.

**Table 3:** Detailed intervention information 2.

No.	EG			CG		
	Intervention	Dose	Frequency	Intervention	Dose	Frequency
1	Acupuncture	—	Once a day for 3 days, then performed every 3 days	Deanxit	Flupirtine, 0.5 mg/meritoxin 10 mg	Once a day
2	Acupuncture	—	Once a day for, 5 times a week	Deanxit	Flupirtine, 0.5 mg/meritoxin 10 mg	Once a day
				Rizatriptan benzoate tablets	1 tablet	If necessary
3	Acupuncture	—	5 times a week	Duloxetine	60 mg/d	Once a day
	Duloxetine	60 mg/d	Once a day			
4	Acupuncture	—	Once a day	TCM	—	Once dose a day
	TCM	—	Once dose a day			
5	Acupuncture	—	Once every 2 days	SSRI antidepressants	—	Once a day for 1 week, then adjust the dosage
	SSRI antidepressants	—	Once a day for 1 week, then adjust the dosage.			
6	Acupuncture	—	5 times a week	Deanxit	Flupirtine, 0.5 mg/meritoxin 10 mg	Twice a day for 10 days, then once a day
7	Acupuncture	—	6 times a week	Amitriptyline	25–150 mg	Once a day

EG, experimental group; CG, control group; TCM, traditional Chinese medicine; —, not available.

**Table 4:** VAS scores in each study.

Author	Year	EG		CG	
		Before therapy mean (SD)	4 w mean (SD)	Before therapy mean (SD)	4 w mean (SD)
Wang	2012	7.0 (1.8)	2.9 (1.0)	6.8 (1.5)	3.2 (1.0)
Ma	2015	7.33 (1.22)	4.81 (1.25)	7.28 (1.19)	5.27 (1.32)
Luo	2018	7.92 (1.16)	2.29 (1.01)	7.86 (1.22)	3.12 (1.25)
Liu	2013	3.68 (1.15)	0.93 (0.78)	3.70 (1.12)	2.53 (1.09)
Cao	2008	7.50 (1.12)	2.34 (1.43)	7.48 (1.27)	2.40 (1.45)

EG, experimental group; CG, control group.

**Table 5:** HAMD scores in each study.

Author	Year	EG		CG	
		Before therapy mean (SD)	4 w mean (SD)	Before therapy mean (SD)	4 w mean (SD)
Wang	2012	18.5 (3.8)	9.3 (3.9)	19.4 (3.4)	10.9 (4.9)
Ma	2015	23.88 (1.86)	16.20 (2.40)	23.91 (1.56)	17.60 (2.29)
Luo	2018	11.09 (2.79)	4.32 (1.41)	11.23 (2.70)	7.06 (2.59)
Liu	2013	24.08 (4.96)	10.84 (3.86)	25.13 (4.96)	14.33 (4.12)
Cao	2008	25.87 (7.76)	12.07 (6.92)	26.43 (9.00)	12.90 (6.01)
Huang	2000	26.71 (5.13)	—	26.87 (4.25)	—

EG, experimental group; CG, control group; —, not available.

**Table 6:** Side effect scores in each study.

No.	Adverse events		TESS score		SERS score	
	EG (%)	CG (%)	EG mean (SD)	CG mean (SD)	EG mean (SD)	CG mean (SD)
1	3 (10%)	15 (50%)	—	—	—	—
2	0 (0%)	4 (16.67%)	—	—	—	—
3	23 (35.94%)	28 (43.75%)	3.25 (1.55)	3.77 (1.86)	—	—
4	7 (16.67%)	9 (21.43%)	—	—	—	—
5	—	—	—	—	3.78 (2.67)	6.48 (4.04)
6	0 (0%)	20 (66.67%)	—	—	—	—
7	—	—	0	10.8 (2.88)	—	—

EG, experimental group; CG, control group; —, not available.

**Table 7:** Assessment of studies' quality.

No.	Randomization	Blinding	Follow-up	Score
1	2	0	1	3
2	1	0	1	2
3	1	0	0	1
4	1	0	0	1
5	1	0	0	1
6	1	0	0	1
7	1	0	0	1

## Data Availability

The datasets used and analyzed during the current study are available from the corresponding author on reasonable request.
